# Optimal Scene Time to Achieve Favorable Outcomes in Out-of-hospital Cardiac Arrest: How Long Is Too Long?

**DOI:** 10.7759/cureus.3434

**Published:** 2018-10-09

**Authors:** Glenn Goodwin, Dyana Picache, Brian J Louie, Nicholas Gaeto, Tarik Zeid, Paxton P Aung, Armando Clift, Sonu Sahni

**Affiliations:** 1 Osteopathic Medicine, Touro College of Osteopathic Medicine, New York, USA; 2 Emergency Medicine, Touro College of Osteopathic Medicine, New York, USA; 3 Emergency Medicine, University Of Miami/Jackson Memorial Hospital, Miami, USA; 4 Internal Medicine, Brookdale University Hospital Medical Center, New York, USA

**Keywords:** scene time, cardiac arrest, ems, cpr, prehospital, rosc, advanced cardiac life support, defibrillation, miami, emergency medicine

## Abstract

Background

Despite advances in resuscitation science and public health, out-of-hospital cardiac arrest (OOHCA) cases have an average survival rate of only 12% nationwide, compared to 24.8% of cases occurring in hospital. Many factors, including resuscitation interventions, contribute to positive patient outcomes and have, therefore, been studied in attempts to optimize emergency medical services (EMS) protocols to achieve higher rates of return of spontaneous circulation (ROSC) in the field. However, no consensus has been met regarding the appropriate amount of time for EMS to spend on scene.

Aim

A favorable outcome is defined as patients that achieved the combination of ROSC and a final disposition of “ongoing resuscitation in the emergency department (ED).” The primary purpose of this preliminary study was to determine the scene time interval (STI) in which American urban EMS systems achieved the highest rates of favorable outcomes in non-traumatic OOHCAs.

Methods

All EMS-related data, including demographics, presenting rhythm, airway management, chemical interventions, and ROSC were recorded using a standardized EMS charting system by the highest-ranking EMS provider on the ambulance. The reports were retrospectively collected and analyzed.

Conclusion

Our data suggest that the optimal 20-minute STI for OOHCA patients in an urban EMS system is between 41 and 60 minutes. Interestingly, the 10-minute interval within the 41-60 minute cohort that provided the highest rate of ROSC was between 41 and 50 minutes. Generally, the longer the STI, the greater the percentage of favorable outcomes up to the 50-minute mark. Once past 50 minutes, a phenomenon of diminishing return was observed and the rates of favorable outcomes sharply declined. This suggests a possible “sweet spot” that may exist regarding the optimal scene time in a cardiac arrest encounter. Significant differences between the average number of interventions per patient were found, however, many confounding factors and the limited data set make the results difficult to generalize.

## Introduction

Out-of-hospital cardiac arrest (OOHCA) is a life-threatening emergency that affects approximately 350,000 Americans a year [1]. Despite advances in resuscitation science and public health, OOHCA has an average survival rate of only 12% nationwide, compared to 24.8% of patients who suffer from cardiac arrest while in hospital [1]. Many factors contribute to favorable patient outcomes in OOHCA, including but not limited to, rapid access to manual cardiopulmonary resuscitation (CPR), early defibrillation, and quick response times by emergency medical services (EMS) [2-4]. There is, however, no consensus on the appropriate amount of time spent on scene by EMS. The scene time interval (STI) is defined as the elapsed time between the responding ambulance arriving on location and when it departs with the patient to the emergency department. Although some prior studies attempted to establish an ideal STI to optimize patient outcomes, nearly all were conducted in Asia [5-7]. The aim of this preliminary study is to identify the scene time interval most associated with positive patient outcomes suffering from cardiac arrest in an urban, paramedic-staffed EMS system in the US.

Miami Fire-Rescue, the EMS system from which the data was retrieved, is staffed by advanced life support (ALS) paramedics [8]. In the United States, paramedics are trained at an advanced cardiac life support (ACLS) level of care for cardiac arrest, including endotracheal intubation, supraglottic airway insertion, intravenous (IV) access, administration of various medications, manual defibrillation, cardioversion, transcutaneous pacing, end-tidal carbon dioxide (CO2) analysis, and blood glucose monitoring [9].

Several of the large studies examining STI took place in East Asia, where EMS protocols and training levels differ. The EMS agencies in these studies operate at the equivalent of a North American EMT-Intermediate/Advanced EMT, which only allows for manual CPR, advanced airway placement, and fluid administration [10]. The different levels of training and resuscitative interventions makes comparison difficult. For example, treatment guidelines in prior studies conducted in other countries recommend much faster scene and transport times compared to Miami Fire-Rescue protocols, possibly due to the differences in available care [6-7]. The increased scope of medication administration and protocols recommending longer scene times directly extend the STI compared to prior studies. Because of this difference, it is important to establish and define the source of this study’s data for consideration.

This study evaluated OOHCA occurring in Miami, Florida, during the year 2016. Miami is a major United States (US) city serviced by the Miami Fire-Rescue Department, a combination fire and EMS service. Miami Fire-Rescue operates 26 advanced life-support ambulances, staffed by two to three firefighter/paramedics [8]. Miami Fire-Rescue responded to approximately 580 cardiac arrest calls in our study period. Data were retrospectively analyzed to determine the rates of prehospital return of spontaneous circulation (ROSC), final disposition upon completion of the encounter, and STIs. A patient was considered to have achieved ROSC if they regained a pulse at any point during the call and was transferred successfully to the receiving emergency department (ED).

Generally, an OOHCA encounter report will only have three different endings: “terminated on scene,” “ongoing resuscitation in the ED,” and “pronounced dead in ED.” Miami’s protocols allow the paramedic to sometimes end resuscitative measures if the patient has obvious signs of death such as rigor mortis or a long downtime (typically longer than 45 minutes). Once the EMS crew arrives at the receiving ED, the ED can continue resuscitative measures or pronounce the patient dead. The most favorable outcome for an OOHCA encounter is for the patient to achieve ROSC and for the encounter to conclude with “ongoing resuscitation in the ED.” The “Favorable Outcome” category in the tables and graphs in this study are those reports that achieved both ROSC and “ongoing resuscitation in the ED.”

This study attempted to determine the STI that produced the highest rates of favorable outcomes while considering other factors that may have influenced them. These various considerations and exclusion criteria are outlined throughout the study.

## Materials and methods

Data collected for this retrospective, observational study was obtained from the City of Miami Fire Department with their expressed written consent. Patient confidentiality was upheld in accordance with Health Insurance Portability and Accountability Act (HIPAA) standards.

Study setting

This study took place in the City of Miami, with an estimated population size of 453,000, during the year 2016 [11]. The 2015 demographics include 75.2% White or Caucasian (including White Hispanic), 70.7% Hispanic or Latino (of any race), 19.3% Black or African-American, 11.4% Non-Hispanic White or Caucasian, 0.9% Asian, and 0.2% Native American or Native Alaskan [11]. The median household income between 2011-2015 was $43,129, with 20% of the population living in poverty [11].

Study population

**Figure 1:**
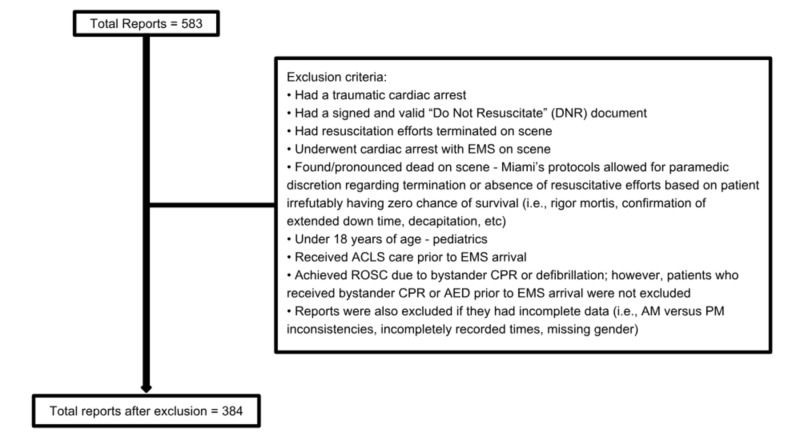
Patient enrollment flow

The focus of this study was centered around patients suffering OOHCA who were treated by the City of Miami Fire-Rescue in the year 2016. The study initially had 583 cardiac arrest reports and following exclusion, 384 patients remained in the final analysis. The 384 patients included 251 males and 133 females of various ethnicities. Patients ranged from 19 to 100 years old. Exclusion criteria and breakdown is provided in Figure 1:

Data collection

All EMS-related data, including patient demographics, chemical interventions (i.e., epinephrine, dextrose), defibrillations, achievement of ROSC, and the conclusion of the case up to the emergency department, was recorded using a standardized EMS charting system by the highest ranking EMS provider on the ambulance. The reports were retrospectively collected and analyzed by the authors of the study. The retrospective nature of this study eliminates a reporting bias. An elaboration of this point is found at the end of the Limitations section.

Data variables

The independent variable of this study was the STI, which included analysis for both 20-minute and 10-minute periods. The dependent variable of a favorable outcome was defined as patients that achieved ROSC at any point during the encounter and were viable enough to continue resuscitation in the receiving ED.

This study also considered the results in the context of chemical and electrical interventions.

Outcome measure

The endpoint of the current study is favorable outcome, a parameter hereby defined as patients that achieved the combination of ROSC and the final disposition of “ongoing resuscitation in the ED.”

ROSC is defined as a palpable pulse in any vessel for any length of time [5]. ROSC attained at any point and for any duration during the scene time interval, in transit to the emergency department, and in the emergency department prior to the dismissal of EMS, were recorded in the study. The focus of this study was purely on the actions, objectives, and environment of the prehospital setting. The objective of every EMS system is to transport patients to the hospital expeditiously while initiating as many integral interventions as possible. With this relatively narrow parameter in mind, factors and trajectories occurring in the hospital setting were deemed as out of scope for this study and, therefore, not addressed. Long-term survivability (months to years post-arrest), neurological capabilities, and subsequent health issues related to the cardiac arrest encounter are some examples of these post-EMS care issues.

Statistical analysis

The values were analyzed using general statistical analysis and percentages. Both the chi-squared test for independence and analysis of variance (ANOVA) with the alpha value set at 95% were used to analyze numerical data.

## Results

Twenty-minute interval cohort

The City of Miami Fire Department responded to 583 cardiac arrest patients during 2016. Of those, 384 cases were included in this retrospective study. As seen in Table 1, the average overall favorable outcome for all cases was 36.98%, which is consistent with the percent ROSC reported in similar studies [12-13].

**Table 1:** 20-minute time intervals Favorable outcome is defined as patients that achieved the combination of ROSC and “ongoing resuscitation in the ED.” The overall percent favorable outcome was 36.98%, which is consistent among each time interval except the 41-60 scene time interval (STI) in which the percent increased to 51.61%. A chi-squared test for independence was used to compare STIs versus outcomes and the differences were not significant, chi-squared (3, N = 384) = 3.11, p = 0.37.

Scene Time Interval	Total Favorable Outcomes	Total Incidents	Percent Favorable Outcomes
0-20	29	80	36.25%
21-40	96	270	35.56%
41-60	16	31	51.61%
61-80	1	3	33.33%
Total	142	384	36.98%

**Figure 2:**
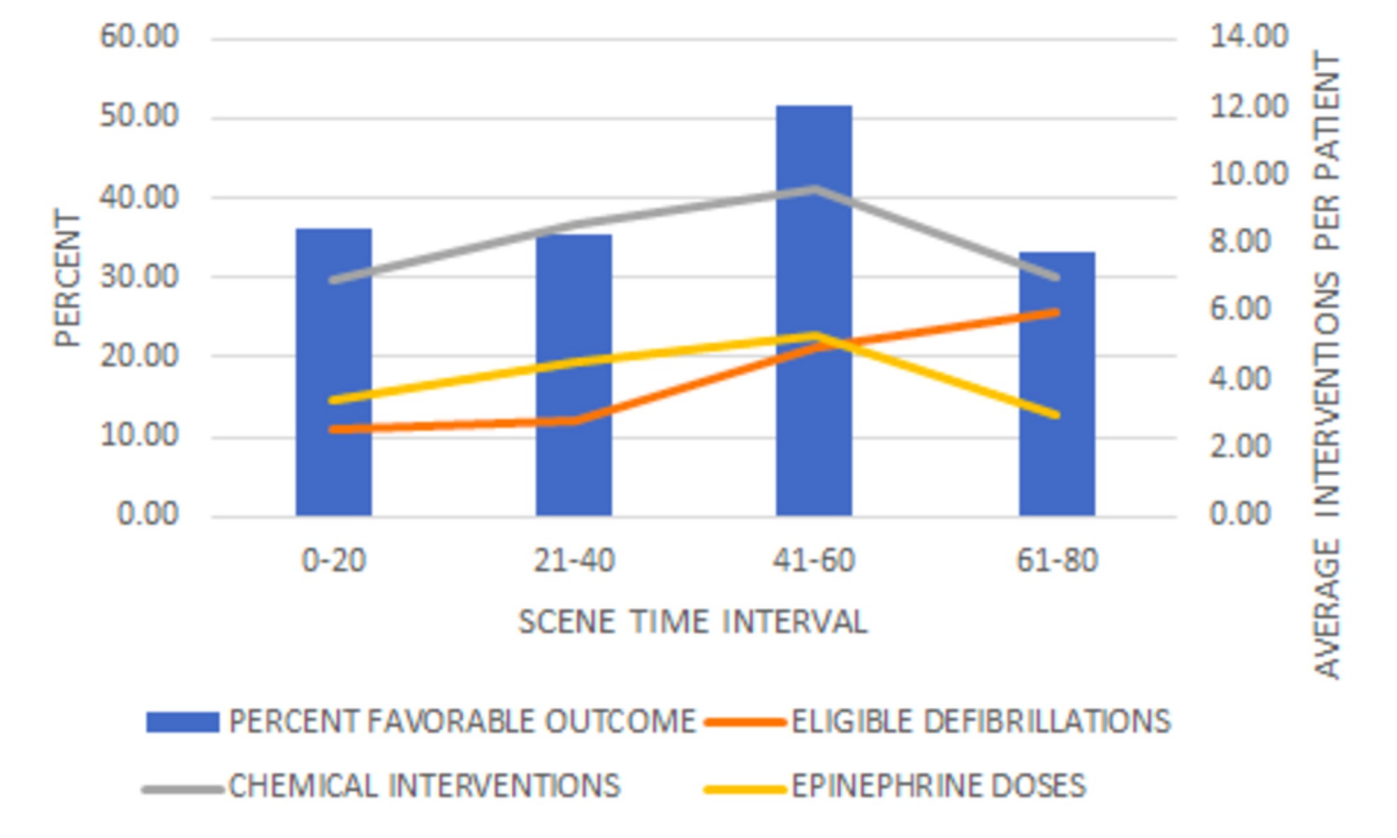
Percent favorable outcome and interventions - 20-minute time intervals Favorable outcome is defined as patients that achieved the combination of ROSC and “ongoing resuscitation in the ED.” No significant differences were found based on STI, F(3, 49) = 1.18, p = 0.32. The average number of chemical interventions per patient in each STI was significant, F(3, 380) = 7.04, p < .001, as well as the average number of epinephrine doses F(3, 380) = 7.8, p < .001.

The cases were divided into intervals of 20 minutes and analyzed according to favorable outcome and interventions administered during the call, as seen in Figure 2. 

Favorable outcome was defined as ROSC plus "ongoing resuscitation in the ED." Interventions included defibrillations and chemical treatments. A patient is considered eligible for defibrillation if the initial presenting rhythm was ventricular tachycardia or ventricular fibrillation without a pulse. Chemical interventions for the purpose of this study were limited to epinephrine administration. In spite of all patients being eligible for chemical interventions, there was one patient who received none at all.

In general, longer STIs correlated with higher averages of interventions, however, it is worth noting that the number of interventions, both electrical and chemical, decreased after 50 minutes. The overall trend of increasing and then decreasing rates of favorable outcomes mirrored the trend of average defibrillations and epinephrine doses.

The number of cases was not evenly distributed throughout the intervals with the majority (270) occurring within the 21-40 minute interval. With the exception of the 41-60 minute interval, the favorable outcome percentages were relatively consistent (Table 1). While the 41-60 minute interval had the highest rate of favorable outcomes, only 31 cases fell into this interval, possibly contributing to imprecision.

Ten-minute interval cohort

In an attempt to determine ideal STI with greater specificity, the data was further subdivided into 10-minute time intervals, with the highest favorable outcome percentage occurring within the 41-50 interval, as seen in Table 2.

**Table 2:** 10-minute time intervals Favorable outcome is defined as patients that achieved the combination of ROSC and “ongoing resuscitation in the ED.” The 0-10 minute STI was eliminated from analysis since there was no data for that period. The highest favorable outcome percentages occurred between 41 and 50 minutes, however, the differences in outcomes among STI was not significant, chi-squared (6, N = 384) = 7.81, p = 0.67.

Scene Time Interval	Total Favorable Outcomes	Total Incidents	Percent Favorable Outcomes
11-20	29	80	36.25%
21-30	59	160	36.88%
31-40	37	110	33.64%
41-50	15	28	53.57%
51-60	1	3	33.33%
61-70	0	1	0.00%
71-80	1	2	50.00%
Total	142	384	36.98%

**Figure 3:**
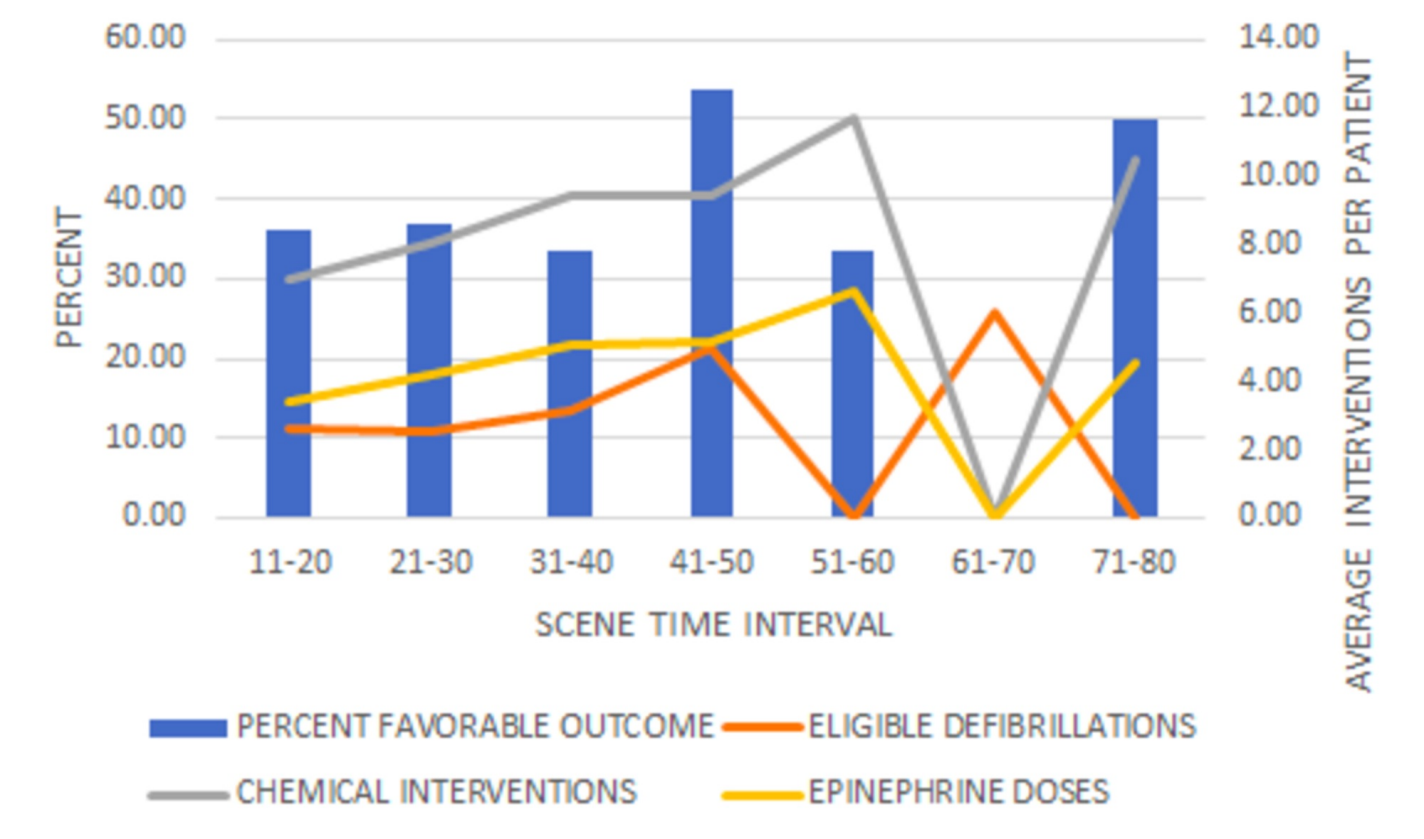
Percent favorable outcomes and interventions - 10-minute time intervals Favorable outcome is defined as patients that achieved the combination of ROSC and “ongoing resuscitation in the ED.” The average number of defibrillations did not significantly differ among STI, F(6, 46) = 0.71, p = 0.64. The average number of chemical interventions per patient in each STI was significant, F(6, 377) = 7.30, p < .001, as well as the average number of epinephrine doses per patient F(6, 377) = 6.4, p < .001.

Similar to the 20-minute STI analysis, the general favorable outcome trend correlated more with average defibrillations and doses of epinephrine, as seen in Figure 3.

There was no observable relationship between interventions and favorable outcome after 60 minutes. An elaboration and implications of this trend can be found in the “Discussion” section.

Statistical considerations

Percentages, chi-squared, and analysis of variance (ANOVA) were used to compare treatments and outcomes across time intervals, however, the differences in percentages can be misleading due to the distribution of data. When analyzed by time intervals, more than half of the cases included in the study occurred between 21 and 40 minutes. Similarly, the data distribution in the 10-minute intervals was not evenly distributed. Because of the smaller data subsets with a skewed distribution, the reported percentages and statistics should be taken into context when making comparisons and drawing conclusions.

## Discussion

The primary purpose of this preliminary study was to explore the relationship between the time EMS spends on scene with favorable patient outcomes. Prior publications studying this relationship were conducted abroad. These foreign municipalities follow vastly different protocols and, therefore, cannot necessarily be generalized to the United States EMS system.

Using data collected from the City of Miami Fire Department, our study demonstrated that the STI correlated with the highest rates of favorable outcome was the 41-60-minute cohort with 51% (Table 1). Within that time frame, the 10-minute interval that correlated with the highest rates was the 41-50-minute cohort (53%).

Despite advanced interventions, such as intubations, IV placement, and drug administration, having uncertain benefits in patient survivability in cardiac arrest, most EMS systems in the United States utilize them [14]. The primary objective of EMS is to transport the patient as quickly as possible to the hospital while simultaneously initiating interventions. Determining the optimal STI facilitates the achievement of this objective by allowing EMS personnel to administer vital interventions while also rapidly transporting patients to the hospital where they can receive more advanced care. Long transport times to the hospital, the capabilities of the EMS personnel and receiving hospital, city layout, and the nature of the incident (hypothermic arrest versus arrhythmia, for example) are just a few of the many factors that go into determining an optimal scene time. An appropriate scene time in a rural setting, for example, may extensively differ from an urban one when considering the aforementioned factors.

One of the biggest determinants of positive patient outcomes is the speed at which a patient receives definitive treatment for their particular ailment [3]. If appropriate treatment can be provided by the EMS personnel on scene, a longer scene time is both reasonable and necessary. In the context of cardiac arrest episodes taking place in an urban pre-hospital setting, many of the initial ACLS interventions can be performed by the EMS responders, possibly accounting for the benefits seen in longer STIs. Having an STI that is too short may not provide the paramedic with sufficient time to administer all necessary interventions prior to arriving at the hospital. By the time the hospital personnel receives the report and performs an assessment of their own, the intervention may not be administered for several minutes more. In addition, the effectiveness of CPR in a moving ambulance is significantly reduced, suggesting that a short STI may lead to poor CPR in transport, which could be harmful to a patient [15-16]. By taking more time on scene to perform quality compressions, end-organ perfusion is increased [17]. This increase in end-organ perfusion may be enough to compensate for the less-than-ideal compressions that can sometimes occur en route to the hospital due to the hindrance of being inside a moving ambulance [16]. Additionally, having more time on scene to circulate the administered medications may also be a positive contributor to patients achieving ROSC and survival [18]. It’s worth noting, however, that long-term survival and functional recovery do not seem to be appreciably improved by epinephrine [18]. The perspective of this study was that of the prehospital EMS provider treating cardiac arrest, which is the time period where epinephrine and other chemical interventions seem to exert their greatest positive effects. The small magnitude of the higher rate of survival and the absence of functional recovery will prompt debate about whether epinephrine is truly beneficial for improving meaningful clinical outcomes [18].

Conversely, having an STI that is too long may allow for the paramedic to perform all the necessary interventions; however, it may also come at the expense of the delivery of more advanced care being only available in the hospital. Serum tests, blood gas values, and radiological assessments are just a few of the numerous examples of these (none can be performed in an ambulance). While our study demonstrates that an intermediate STI value of 41-60 minutes is the most ideal, one must consider various factors that may hinder the generalizability and possible clinical implications of our findings.

Other studies have demonstrated optimal scene times as being much lower (in the 10-30-minute range), however, those studies had vastly different influences and metrics [5-6]. Most of the previous studies took place in a demographically homogeneous setting with a more limited array of different ethnicities as compared with the current study. Many of them were conducted in South Korea, Japan, or Canada [5-6]. As previously stated, the highly diverse population of the study population in Miami carries a myriad of different medical conditions, many of which directly influence patient survivability and treatment. Rates of hyperlipidemia, chronic kidney disease, diabetes, and heart disease are just a few of the medical conditions that vary across races and ethnicities [19-20]. In addition to differences in patient demographics, there are differences in the training and capabilities of the EMS personnel. For example, a typical Korean EMS ambulance is staffed by an EMT-intermediate, an EMT-Basic, and a driver [7]. As briefly touched on in the Introduction section, Miami Fire Department ambulances are equipped with three ACLS-certified paramedics. Paramedics have a significantly expanded scope when compared to EMT-B or EMT-I level responders [9]. Additionally, paramedics are trained in performing many other tasks, such as endotracheal intubation, IV drug administration, cricothyrotomy, and pleural decompressions. The additional education and capabilities seen in the Miami Fire Department’s EMS system may impose a considerably longer STI than other EMS systems. Furthermore, the metrics of positive patient outcomes varied across the studies. The data limitations of our study prevented the utilization of more conclusive survivability outcomes, such as neurological recovery and absolute survivability. Many of the previous studies that explored this subject employed those considerations, thereby making their conclusions more definitive, and possibly contributing to the vastly different ideal STI values [5-7]. It is important to note that one study from Japan concluded that STIs of at least 40 minutes correlated with both, the highest rates of ROSC as well as the best neurological outcomes [21]. The diversity of optimal STI figures seen in other studies and this one exemplifies the point that many factors comprehensively contribute to the individuality of a municipality’s ideal STI.

Limitations

In addition to eliminating the reporting bias, the retrospective nature of this study also precludes the determination of causality. The researchers also did not control for any confounders, such as patient age, which could appreciably impact the aggressiveness of treatment. Generally, a much older patient is regarded as less viable and may not always receive the same amount of cardiac arrest interventions. The reasons for this age discrepancy are numerous and not fully understood but the cardioprotective effects of estrogen in premenopausal women seem to be the most compelling [22-24]. Moreover, the exclusion criteria eliminated 199 patients, almost all of which did not achieve ROSC, which may inflate the rates seen in this study

Our analyses were limited to the City of Miami, FL, and the Miami Fire-Rescue Department. The population density, city layout, and training of EMS personnel can vary greatly across municipalities, thereby making the obtained results less generalizable [25]. Miami’s racial demographics differ from the US average: a significantly higher percentage of Hispanics (67.7% in Miami vs 17.8% nationally) and a higher percentage of African Americans (18.5% vs 13.3% nationally) are two of the more notable differences [11,26]. The racial makeup of a city is an important consideration because arrest outcomes can significantly vary among different races and socioeconomic statuses, and certain cardiovascular diseases are seen at different rates between ethnic groups [19-20,27]. Interestingly, the delivery of care from bystanders seems to vary across the races and socioeconomic statuses of the patients receiving the care, which could also affect outcomes [25].

The data was only based on the self-reported accounts of cardiac arrest episodes by the paramedics prior to hospital arrival. While all reports that contained obvious reporting errors were eliminated from our dataset, they were all written in the uncontrolled environment of the prehospital setting. Furthermore, reports were only written and edited by the lead paramedic, so it is possible that other errors were not taken into account.

The number of patients who went on to survive for a prolonged period of time following ROSC was unable to be obtained, preventing one from drawing comprehensive conclusions regarding true survivability. However, the focus of this study was purely on the actions, objectives, and environment of the prehospital setting. The objective of every EMS system is to transport patients to the hospital expeditiously while initiating as many integral interventions as possible. With this relatively narrow parameter in mind, factors and trajectories occurring in the hospital setting were deemed out of scope for this study and, therefore, not addressed. Long-term survivability (months to years post-arrest), neurological capabilities, and subsequent health issues related to the cardiac arrest encounter are some examples of these post-EMS care issues. While not rigorously studied, the authors did have access to the Ustein Survivability Report, which showed the number of patients that went on to be discharged, alive, from the hospital. Determining which particular patients survived to discharge from the hospital was not possible, therefore, the analysis of that report was limited. These numbers are provided under the Clinical Implications and Recommendations section.

Other positive patient parameters, such as end-tidal CO2, were not utilized in this study, further limiting generalizability regarding patient outcomes. The researchers were also limited by incomplete data on bystander CPR, patient ethnicity, patient downtime prior to EMS arrival, and patient medical history.

There were intrinsic limitations to using the scene time interval. STI is the elapsed time starting from the moment the ambulance arrives on scene up to the time the ambulance begins transporting the patient to the hospital. It does not account for the time required for the EMS crew to access the patient (vertical response time) [28]. Various factors, such as being in a large building, having difficulty entering a premises, responding to a crime scene, are just a few of the many circumstances that routinely prolong vertical response time. Treatment errors or limitations such as having difficult IV access, failed intubations, and challenges in moving the patient were not taken into account and would artificially prolong the STI.

While a retrospective study brings many intrinsic limitations, all of which were discussed in the aforementioned paragraphs, it also brings some advantages; the most compelling of which is the accuracy of outcomes. To elaborate on this point, consider the following: paramedics were unaware that their actions would be analyzed and used for a future study. Had they known that they were being researched, as in the case of a prospective study, they may have acted differently, thereby skewing the results accordingly. By going back after the fact and seeing which actions naturally occurred, the conclusions drawn are more reliable. Just as a paramedic’s actions could have differed if they knew they were being researched, this phenomenon also extends to the reporting practices as well. Analyzing the reports after the fact prevented the bias or adjustments that could have reasonably been made, thereby facilitating the practical accuracy of the results.

Clinical implications and recommendations

STIs may be a useful predictor of positive patient outcomes in cardiac arrest victims, however, the specificity of Miami’s environment and demographics prevents one from drawing definitive and generalized conclusions. Additionally, the utilization of ROSC achievement as a positive patient outcome metric is limiting because a large number of patients who achieve ROSC do not end up surviving. According to Miami’s Utstein Survival Report, of the 125 patients who achieved sustained ROSC, only 26 were successfully discharged from the hospital. What constitutes “sustained ROSC” is not clear, which is why this study chose to include ROSC of all durations.

Although the results of this study are not generalizable, the correlations seen in various STIs are significant enough to prompt further research in this field. To draw more decisive conclusions, future studies must be conducted in a wide array of municipalities and environments and utilize more comprehensive metrics of cardiac arrest success. By definitively determining a comprehensive set of optimal STIs for given medical conditions, EMS protocols can be adjusted accordingly, leading to better patient outcomes.

## Conclusions

Our data suggest that the optimal STI for favorable outcomes after OOHCA for patients in the study population is between 41-60 minutes. More specifically, the 10-minute interval within the 41-60 minute cohort that provided the highest rate of favorable outcome was 41-50 minutes. Favorable outcomes increased in our dataset until approximately 50 minutes. Once 50 minutes was reached, a phenomenon of diminishing returns was observed and the rates of Favorable Outcomes sharply declined. While the aforementioned correlation was merely a trend (not statistically significant), a statistical significance in the average number of chemical interventions and epinephrine administered was noted. In general, the observed trends followed the logical correlation that more time on scene equals more interventions, both electrical and chemical, all of which peak at around 41-50 minutes. As with the rates of favorable outcomes, the expected trend almost completely dissipates after 50 minutes, perhaps suggesting that resuscitative efforts are critical up until a certain STI after which more advanced interventions are necessary.

Many confounders contributing to patient outcomes and STIs were not controlled for, therefore, the results are not generalizable. Further studies with larger data sets are necessary to truly explore the impact of scene time on patient viability as well as the relationship between STI and interventions.
